# Early innovations in maritime telemedical services: the KDKF Radio Medico Station

**DOI:** 10.5195/jmla.2023.1567

**Published:** 2023-04-21

**Authors:** Johnathan Thayer, Stefan Dreisbach-Williams

**Affiliations:** 1 jthayer@qc.cuny.edu, Assistant Professor, Graduate School of Library and Information Studies, Queens College, City University of New York, Queens, NY.; 2 stefan.dreisbachwilliams@seamenschurch.org, Archivist, Seamen's Church Institute, New York, NY, previously Maritime Associate at the Waterfront Alliance, Registration Coordinator at the Center for Wooden Boats, Seattle, WA.

**Keywords:** Telemedicine, radiotelegraphy, seafarers

## Abstract

“MAN PUT HIS TONGUE AGAINST REFRIGERATOR PIPE AND GOT IT FROZEN; HAVE THAWED IT OUT AND IT IS NOW BLISTERED AND SWOLLEN BUT NOT PAINFUL. ARRIVING HONOLULU FRIDAY; HOW CAN I HELP HIM MEANWHILE?” Thus read a message relayed via radiogram across the ocean to the physician stationed at the Seamen's Church Institute's (SCI) KDKF radio station, established by the Institute in 1920 on top of its thirteen-story seafarer services center at the southern tip of Manhattan. Though radio was in its infancy, radio telegraphy had already proven its revolutionary power, featuring prominently in far more serious maritime emergencies such as the sinking of Titanic. SCI's KDKF radio station aimed to address a less dramatic but no less important problem in blue water navigation: access to medical care.

**Figure 1:**
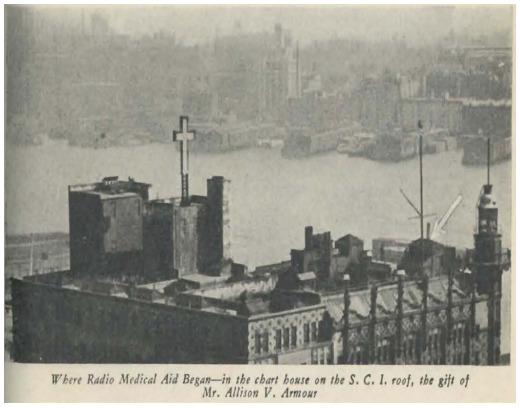
“Where Radio Medical Aid Began–in the chart house on the S.C.I. roof” [24]

## INTRODUCTION

The extreme conditions of isolation under which seafarers work and live has prompted a long timeline of changes in information and communication technologies related to seafarer services, including medical care. The Seamen's Church Institute (SCI), which has worked to improve conditions for seafarers since 1843, developed and operated KDKF Radio Medico as an unequivocal innovation in maritime medicine that continues to influence efforts to address seafarers' health and safety needs today.

The case study of the Seamen's Church Institute's (SCI) KDKF Radio Medico Station not only provides insights into early innovations in maritime telemedical services; it is also situated within a larger timeline of initiatives and motivations tied to the foundations of United States maritime reform and navalism. Specifically, KDKF and other maritime health and wellness initiatives of its era can be thought of as linked to the doctrine of “flexible maritime capacity,” which maintained that a stable and healthy citizen merchant marine was essential to national security interests by providing an auxiliary to the military in times of war and imperial expansion. Flexible capacity derived from Mahan's *The Influence of Sea Power Upon History* and was endowed with greater urgency by imperial projects embedded in the Spanish-American War, and later, the First and Second World Wars [1, 2].

Additionally, this case study extends knowledge about the history of medical information delivery via radiogram and documents early innovations in telemedicine. The unique health information needs of maritime workers during the early 20th century provide context for current issues and innovations in telemedicine, especially during an era of pandemic and the increase of remote health services.

### Historical Background

Founded in 1834 and still in operation today, the Seamen's Church Institute (SCI) is dedicated to improving the welfare of merchant seafarers both aboard and ashore. Early initiatives in the nineteenth century involved bringing religious services closer to mariners with floating churches and reading rooms along the waterfront of lower Manhattan. These served as outposts for temperance campaigns and efforts to combat coercive hiring practices.

In 1913, SCI centralized its operations to a thirteen-story mariner services headquarters at 25 South Street on Coenties Slip where canal boats and steamers from inland waterways moored beside ocean-going freighters just a few blocks from the Staten Island Ferry. 25 South Street offered mariners cheap rooms and a cafeteria, employment services, tailor and barber shops, a chapel, a soda fountain, and more [3].

In 1925, SCI established a Medical Clinic on the mezzanine of 25 South Street staffed by members of the Public Health Service. Ear-nose-throat, dental, and eye clinics followed, and by 1931 25 South Street had supplemented the religious and philanthropic foundations of its mission to serve as a sort of unofficial auxiliary to the US government by providing services to maintain the health and preparedness of the nation's merchant maritime labor force [4].

Following the market crash of 1929, the federal government enlisted private charities like SCI to provide economic relief, allocating funds to the Institute to be distributed to destitute seamen who were clogging port cities' boarding houses and relief houses alike during the years of the Great Depression. The maritime ministry project that SCI administered during the 1930s served a nationalist economic role in stabilizing an urban labor force that was experiencing the shockwaves of unemployment and that was being recruited into an increasingly radical front of organized labor. Additionally, acting within the doctrine of “flexible capacity,” SCI provided a space in which mariners could be housed and prepared for service as an essential arm of the US national security apparatus. SCI's auxiliary relationship to US national security was made explicit when the US entered World War II, and SCI was called upon to serve as an official training and reserve station for the nation's rapidly expanding merchant marine. The fact that President Franklin D. Roosevelt had served on SCI's Board of Managers since 1907 when he was Assistant Secretary of the Navy only strengthened the connections between 25 South Street and the US military [5].

The economic and geopolitical roots of SCI's role in providing for the health and wellness of merchant seamen began much earlier than the tumultuous 1930s. By the end of the nineteenth century, SCI had added education to its services. The notion that civilians should receive formal basic medical training had been adopted broadly in the 1870s and SCI had aggressively sought to teach first aid to sailors since 1910. The goal was to ensure that all officers had enough training to provide on the spot care and assist a ship's doctor. The institute had struggled and ultimately succeeded in ensuring that all ships had a medical chest aboard, as required [6]. Getting doctors aboard cargo ships was a harder sell. In 1921 more than 75% of ships at sea had no doctor aboard, but more than 80% did have radios [7]. Unreliable access to medical information at sea proved to be yet another risky aspect of an already dangerous occupation. If an illness or accident struck a sailor at sea, they were entirely dependent on the knowledge and expertise of anyone who happened to be on board. As SCI bluntly described this arrangement, “If there is no one who can help, it is a race between the vitality of the patient and the injury or sickness. If he gets better he gets better and if he dies, well he dies” [7]. Connecting seafarers with doctors via radio would radically reduce the isolation from medical information and mitigate the perils of illness and injury at sea.

During WWI the education programs housed in the top floors at 25 South Street expanded significantly with the increased need for seafarers as they were called into the war effort. SCI's navigation school added to the first aid training such topics as knot tying, navigation, lifeboat handling, and semaphore. SCI credits Captain Robert Huntington, Principal of SCI's Navigation, Marine Engineering, and Radio School with the idea of connecting ships at sea with doctors on land via radio, which came to him after hearing a message from fog-bound ships on his receiver as they tried to determine their positions [8].

### KDKF Radio Medico

Radio telegraphy was an attractive addition to SCI's education offerings but required substantial financing to acquire the necessary equipment and power the signal. A gift of $5,000 from steel magnate Henry A. Laughlin fitted out a small room in 25 South Street's tower with all the equipment required to produce a radio telegraphic signal and receive transmissions [8].

In 1920 SCI launched what arguably remains to this day its most innovative and influential program: a radio service that connected doctors on land to ships at sea. This service was so successful that it was soon adopted by the federal government in cooperation with the Radio Corporation of America, and similar operations sprang up around the world. Maritime Telemedical Assistance Services (TMAS) throughout Europe and North America trace their roots to this project [9].

Radiotelegraphy, which transmits beep tones rather than articulated sounds or speech, was first demonstrated in 1901. In the following two decades, radio exploded in application. The Wireless Ship Act of 1910 required all ships carrying more than fifty passengers more than two-hundred miles off the coast to carry radio equipment with a range of one-hundred miles. The Radio Act of 1912 required all seafaring vessels to maintain a twenty-four-hour radio watch. Under these circumstances, SCI's radio medical services seem both innovative and inevitable.

Henry A. Laughlin's check for SCI's radio equipment, viewable on a lantern slide in SCI's archives, has a date of December 1920 but SCI applied for a radio license when the US government initially made them available. The first radio station license in America was issued on October 27, 1920. One week later, SCI received license no. 176. The service was assigned the call letters KDKF, and the MEDICO call sign was given priority over every other call except SOS. If a ship couldn't reach KDKF directly it could reach out to a ship located closer to New York in case that ship had a doctor on board. If it didn't, it could pass the message on to additional ships as necessary till a chain of radio operators had connected the ship in need of medical attention to either a doctor on board another ship or the doctor on call through KDKF. SCI provided the service free to ships regardless of nationality [8].

Initially KDKF's license only allowed it to operate from 9am-5pm, which is when SCI's doctor was on duty in the clinic. During hours outside of KDKF's operation, ships and seafarers were again left on their own, as is documented in SCI's archives:

“One poor unfortunate fellow, was taken ill while our doctor was not on duty, and the call for advice was not answered. He died, and we cannot but feel that had we been able to keep a doctor on duty all the time, his life might have been saved” [10].

In short order, the Hudson Street Hospital (located about 1.3 miles north of 25 South Street) offered to have a doctor available to SCI by phone at any hour, and SCI's license was expanded to twenty-four-hour transmission in April 1921 with service beginning in May. By 1922 KDKF radio operators were contacting doctors at the Public Health Service Hospital No. 70 on the other side of the harbor at Staten Island for responses to medical and surgical requests [11].

**Figure 2:**
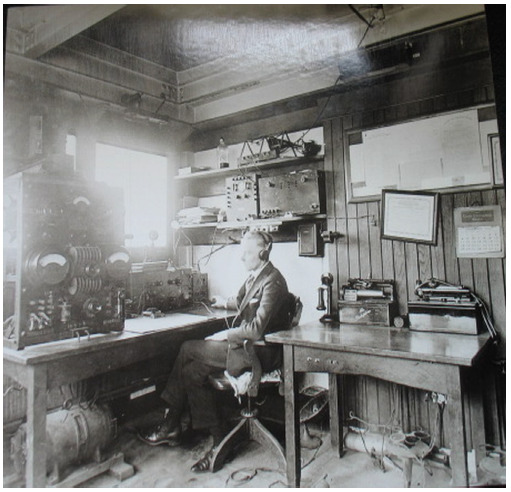
Wireless station on the roof of SCI's headquarters at 25 South Street [24]

**Figure 3:**
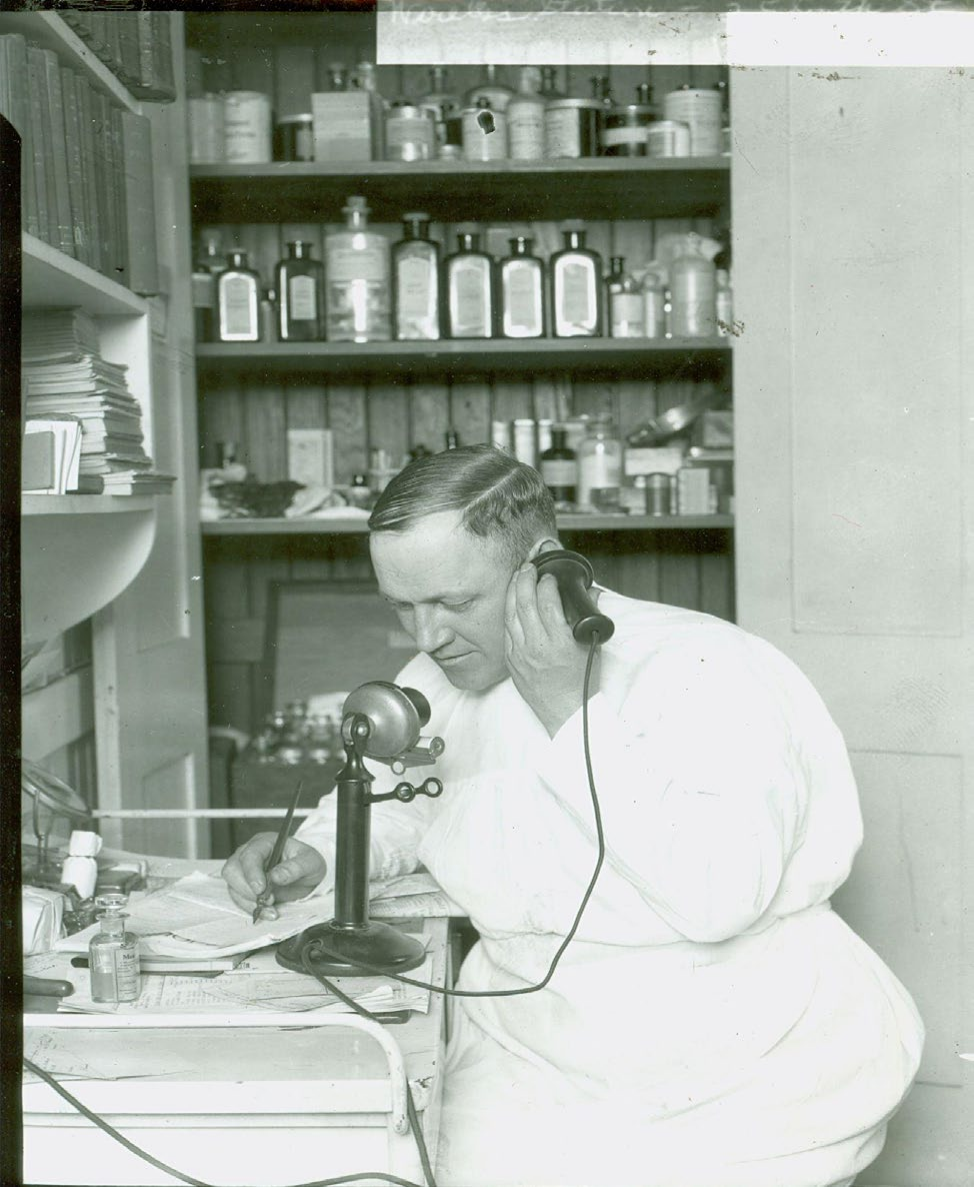
Medical information is relayed via SCI's KDKF Medico service [24]

KDKF's success relied not only on connecting doctors with ships' crews, it also required those crews to have training to properly implement that advice. SCI required young officers enrolled at its Navigation and Marine Engineering School to learn how to care for the sick and wounded before receiving their certificate. As part of this training they learned how to communicate effectively with a doctor via radio, concentrating on unambiguous proper terms.

SCI augmented the radio service with its Manual on Ship Sanitation and First-Aid for Merchant Seamen, first published in 1922, to provide much-needed medical resources for merchant ships. Devoted to care of the ship and self, SCI's Manual served “to meet one of the greatest humanitarian needs on board our Merchant Vessels” with a quick reference for medical and surgical conditions risked at sea as well as directions for disease treatment [12].

KDKF contributed to SCI's education work in other ways as well because the radio equipment gave sailors the opportunity to learn radio operation, however SCI's involvement with radio medicine was short-lived. By 1922, the Radio Corporation of America had offered to take over and expand the service. The signal moved from 25 South Street to a tower at Bush Terminal in Brooklyn and by March 1922 KDKF was offline [13]. By 1923, as reports reached 25 South Street of new radio telemedical services in Norway and Sweden, SCI declared its intention to preserve the KDKF radio equipment, which was no longer in use [14, 15].

In addition to providing guidance for dealing with occupational injuries like a dislocated shoulder: “LAY THE SAILOR FLAT ON DECK. TAKE SHOW OFF AND PLACE YOUR HEEL IN THE ARMPIT OF THE SAILOR. GRASP THE HAND OF THE DISLOCATED ARM AND PULL OUTWARDS SLIGHTLY,” and injuries on passenger ships: “FIFTEEN YEAR OLD GIRL SWALLOWED SAFETY PIN OPEN IN THrOAT. BOAT IS NOT EXPECTED TO DOCK UNTIL 3:30 P.M. CAUSING CONSIDERABLE PAIN PLEASE SEND INFORMATION,” expanded MEDICO services were able to assist with the control of contagious disease. In 1936, MEDICO was able to connect the Grace liner *Santa Panla* with the California State University Maritime training ship, which was overrun with a meningitis outbreak among the cadets. The *Santa Panla* rerouted and met the training ship in order to provide medical supplies and prevent further spread of the disease [16].

SCI's 25 South Street was demolished in 1967, and it's unknown what became of its radio equipment, but radio medicine remains a vital service to ships at sea. Today's Maritime Telemedical Assistance Service organizations continue the work started with KDKF. New technologies like Telex have come and gone from the TMAS landscape while radio and VHS have only recently been supplanted by satellite phones and email with digital photos [17]. TMAS services have been cemented into maritime practices through international agreements, notably the International Labor Organization on Health Protection and Medical Care (Seafarers) of 1987 [18].

In fall of 2020 the Centro Internazionale Medico (International Center of Medical Radiocommunications) hosted a round table (held virtually, in keeping with the theme and the times) commemorating “100 Years of Radio/Tele Medical Assistance at Sea,” with presentations on the modern applications that trace their roots to KDKF. These applications use all the current groundbreaking technologies with digital databases accessible via satellite to reduce the isolation that heightens the risk of seafaring [19, 20].

In the intervening century, SCI has built on its success in improving medical conditions at sea by addressing mental health concerns that are unique to seafarers. SCI has led the effort to understand the effects of piracy on seafarers, publishing “Guidelines on Post-Piracy Care for Seafarers” [21]. The Institute conducted shore leave studies in order to survey seafarers' access to resources and services off ship in US ports, and has most recently met the challenges posed by COVID-19-related restrictions to mobility in port [22]. And maritime ministry organizations on an international level have conducted information needs assessment studies to gauge seafarers' priorities in combating the effects of isolation at sea through communication technologies [23].

Clearly, SCI's early health and wellness initiatives, marked by the innovative extension of services to the high seas through emerging radio technologies more than a century ago, continue on through to the present day. Whereas this paper has attempted to contextualize such initiatives within their historical context during the late-nineteenth and first half of the twentieth centuries within the naval and geopolitical theory of flexible capacity, current health initiatives involving a merchant maritime fleet and labor force that is increasingly foreign in both flag and crew might be more closely linked to motivations rooted in an ever-expanding global economy dependent on maritime shipping.
